# Longitudinal patients' cohorts for impactful research and HIV care at the Infectious Diseases Institute

**DOI:** 10.4314/ahs.v22i2.4S

**Published:** 2022-08

**Authors:** Barbara Castelnuovo, Moses Kamya, Rosalind Parkes Ratanshi, Agnes N Kiragga, Damalie Nakanjako

**Affiliations:** 1 Infectious Diseases Institute, College of Health Science, Makerere University; 2 College of Health Science, Makerere University

**Keywords:** Longitudinal Cohorts, Uganda, HIV

## Abstract

**Introduction:**

Observational studies provide important evidence supporting the feasibility and effectiveness of health interventions. The 20-year-old Infectious Diseases Institute (IDI) established to respond to infectious diseases, specifically HIV/AIDS, invested in the set-up of longitudinal cohorts. In this paper we discuss the results of these cohorts and their impact on the response to the HIV pandemic in Uganda.

**Methods:**

IDI invested in experienced system developers, clinic and laboratory capacity to create the infrastructure to host longitudinal cohorts. Several cohorts were created, including patients initiated and followed up on ART, specialized cohorts (e.g. TB co-infection) and long-term cohorts with patients on ART for over 10 years and aged 60 and above. These cohorts function as platforms for sub-studies, attracting collaborators and students.

**Results:**

Data from the IDI cohorts contributed evidence to ART programs on when to start, which drugs to use, how to best monitor and which models of care to implement. Sub-studies contributed to management of opportunistic infections, understanding immunological response and the emerging complications of non-communicable diseases.

**Conclusion:**

Cohorts provide a platform for clinical care, training, and research to inform strategic responses and put Makerere University at the center of the response to the HIV pandemic in the region.

## Introduction

Makerere University's Infectious Diseases Institute was established 20 years ago within the College of Health Sciences (https://idi.mak.ac.ug/) as strategic initiative in the response to the HIV/AIDS pandemic through comprehensive HIV care including antiretroviral therapy (ART-for people living with HIV (PLWH), research and training. In order to understand HIV/AIDS disease within the local context, the IDI alliance of Ugandan and American scientists, The Academic Alliance, found it relevant to set up longitudinal cohorts. By design, cohort studies are meant to be more inclusive^1^, ^2^ (e.g. patients with comorbidities, older, sicker) as compared to randomized controlled trials (RCT) which remain essential to investigate the safety and efficacy of drugs or intervention, although RCTs may not provide a real world picture of how patients interact with medication and other interventions^3^. Longitudinal cohorts follow up participants for longer periods^4^, are designed to detect events that were not anticipated by the results of RCTs^2^, and are hypothesis generating rather than hypothesis driven^5^.

Though costly to set up and maintain for the long period, longitudinal cohorts provide important additional evidence supporting the feasibility of scaling up treatments or interventions, their effectiveness^6^, and continue to generate insights into the challenges and benefits of implementation of health interventions across different settings and populations^7^. Cohort studies provide relevant context specific data^8^; for example, cohorts of people living with HIV in resource rich countries may not be applicable to sub-Saharan Africa (SSA) due to differences in populations, demographics, laboratory and human resources, and epidemiology of opportunistic infections. The main limitations of using routinely collected observational data for research are missing information, confounding by indication, failure to assess causality, especially data collected in busy clinical settings with limited number of health care workers. To address this gap in the IDI HIV clinic, well characterized cohorts were set up to ensure that observational data is systematically collected, and desirably in more detail, for a longer period^12^.

This paper discusses the concept, investment and impact of setting up cohorts that are relevant to understand relevant clinical care interventions and outcomes of patients on ART in SSA, and how the relevant evidence and publications from the cohorts have contributed to HIV care policies and practice in the SSA region in answering questions such as: 1) When to start? 2) Which drugs to use? 3) How to best monitor? 4) How do patients respond to treatment immunologically? 5) Which models of care? 6) How are co-morbidities managed?

## Methods

### Setting

IDI is a center of excellence^13^, integral part of the School of Medicine within the College of Health Sciences, Makerere University, for HIV treatment and prevention. The IDI adult clinic, located within Mulago Hospital with over 30,000 patients ever registered, was one of the first center in the country to receive antiretroviral therapy (ART) for routine HIV care and treatment; therefore, this presented a unique opportunity to set up early cohorts of patients starting ART to inform short and mid-term outcomes. Anti-retroviral drugs have been provided by the Global Fund and the President's Emergency Plan for AIDS Relief starting in 2004. ART is prescribed and monitored according to the prevailing WHO and/or Uganda Ministry of Health guidelines^14^–^18^.

### Infrastructure

The IDI site at Mulago provides an ideal setting to conceive and implement longitudinal cohorts set up with the aim of understanding the challenges and successes of the scale up of ART un Uganda.

**Figure 1:**
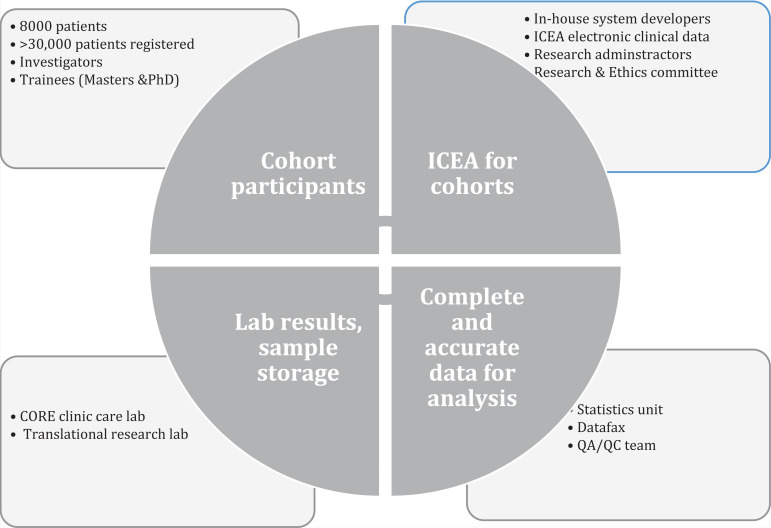
Systems at IDI which enable the successful implementation of longitudinal cohorts

IDI invested in experienced system developers, and data collection tools and electronic medical records were designed and implemented in house. The Integrated Clinic Enterprise Application (ICEA) was developed in-house as a provider-based entry with an important planned feature to reduce the rate of errors, provide real time validation of data, and automate tasks such as drug prescription writing 19. IDI hosts a Core Laboratory (previously Makerere University-John Hopkins University (MUJHU) Core Laboratory) which is certified by the College of American Pathologists to perform routine blood tests (including hematology, biochemistry, HIV viral load). Biological samples including plasma, serum, peripheral blood mononuclear cells and others can be processed and stored by the IDI research translational laboratory, which is an in-house research lab for mycology, immunology, and molecular biology assays as required by the respective studies. Furthermore IDI established a research program in 2005 which includes a data Quality Assurance and Quality Control (QA/QC) team and a statistical unit specialized in the analysis of longitudinal data. All these resources contributed to the successful implementation of the early cohorts (Figure 1).

### Cohorts at IDI

Two cohorts were started in 2004, the IDI Research cohort (559 patients starting ART) and the IDI Routine cohort including all clients ever registered at the clinic. These cohorts aimed to describe outcomes of PLWH starting ART. With time the IDI clinic shifted due to the experience of the health care provider to become a specialized setting where more complex patients can be up-referred, while stable patients on ART are down-referred to health care settings near their residence. Thus permitted the implementation of specialty clinics and data collection and analysis sub-cohorts (e.g. outcomes of pregnant women on ART and their babies), and more formal research cohorts were formed, particularly the Geriatric cohort (adults≥ 60 years), and the Post TB cohort^20^.

Finally since a great number of IDI patients started ART during the early scale up of ART and successfully lived up to 10 years on ART, 1,000 patients were enrolled in the long term ART cohort to understand HIV in the elderly^21^. The various IDI cohorts and detailed description are presented in Table 1.

**Table 1:** The Infectious Diseases Institute cohorts' description

	Research cohort	Routine cohort*	Post-TB cohort	Long term cohort	Geriatric cohort
Patients	559 starting ART	All patients registered at IDI (<30,000)	203 patients completing TB treatment	1,000 on ART for 10 years	500 on ART
Follow up Visits	10 years Every 3 months	Indefinitive All clinic visits at IDI	5 years All clinic visits at IDI	10 years Yearly	2 years Yearly
Data collected	ART, toxicity, adherence (3 scales), OIs	ART, toxicity, adherence, OIs	ART, toxicity, adherence, OIs	ART, toxicity, adherence, OIs, history of NCDs, co-medication	Same as long term cohort plus screening for NCDs and geriatric syndromes
Blood tests	Hematology, biochemistry, CD4 count, viral load	As per routine at IDI according to current guidelines	As per routine at IDI according to current guidelines	Hematology, biochemistry, CD4 count, viral load	Hematology, biochemistry, CD4 count, viral load
Data collection	ICEA research cohort	ICEA routine clinic	ICEA routine clinic	ICEA long term cohort	ICEA long term cohort RedCap
Sample storage	Plasma	None	Sputum, cells	Cells, plasma, serum	Cells, plasma, serum
Status	Closed	Open cohort	Closed, under analysis	Follow up	Enrolment completed

* Includes specialized cohorts

ART: antiretroviral treatment; IDI: Infectious Diseases Institute NCDs: non communicable diseases; ICEA: Integrated Clinic Enterprise Application

## Results and impact of the results

### Data analysis results of the IDI research cohorts

Table 2 presents some of the published results generated from the data analysis of the 2 fully implemented cohorts (Research cohort and Routine cohort).

**Table 2:** Results generated from the data analysis of the Research cohort and Routine cohort

	Research cohort	Routine cohort
**When to** **start**	High mortality^22^ and occurrence of opportunistic infections^23^ in patients started on ART with low CD4 count	Incident TB during ART occurs most often within 3 months and in patients with low CD4 counts and it associated with long-term impairment in immune recovery^24^
**Which** **drugs**	Drug toxicity in patients starting ART during the scale up of ART is driven by stavudine toxicity in women^25^ Good virological response but high rate of toxicity with second-line antiretroviral therapy^26^ Patients on zidovudine-containing regimen are more likely to develop suboptimal immune reconstitutions compared to those on stavudine^27^	High mortality in patients on stavudine with lactic acidosis^28^ Toxicity as reason for drugs substitution decreased over time mirroring the phase out of stavudine^29^ Dolutegravir-associated hyperglycaemia in patients with HIV^30^
**How to** **monitor**	Reappearance of skin manifestation are suggestive of virological failure 31 The use of at least one repeat measurement rather than a single VL measurement could avert from 60% to 80% of unnecessary switches^32^ Development of algorithms based on clinical and immunological criteria to improve WHO criteria^33^	High rate of misclassification of treatment failure based on WHO immunological criteria^34^ Delay in switching patients with treatment failure confirmed by ad hoc viral load^35^ Delays in repeating viral loads in patients with suspected treatment failure after implementing routine viral monitoring 36
**Models of** **care**	Close follow up and adherence counselling result in low lost to follow up, good immune response and viral suppression^37^ The long term virological outcomes in a specialized HIV treatment center are comparable to those from research-rich settings^38^	Task shifting improves patients flow, reduces waiting time^39^ and it is cost effective^40^, ^41^ Integration of HIV and TB services results in improved TB treatment outcomes and prioritized ART initiation^42^ Very low documented HIV transmission comparable with those reported in clinical trials setting^43^

TB: tuberculosis; ART: antiretroviral treatment; WHO: World Health Organization;

Additionally, the IDI cohorts have served as platform for other sub-studies attracting local and international collaborators and students. The methods and key results of the sub-studies are presented in Table 2.

### Lessons learnt

Cohorts remain an important resource to inform programs at national and international levels by providing critical information on population outcomes in real world settings, outside the controlled setting of randomized trials.

IDIs investment in longitudinal cohorts developed from a simple cohort of patients starting ART to several more specialized cohorts answering different questions, as shown in Table 1. Over this time period the investigators have gained considerable experience in study design, collection tool development, and statistical analysis consistent with international standard. Though costly to set and maintain, these cohorts are the cornerstone to IDI's success in conducting impactful multi-disciplinary research to understand the HIV pandemic over the last 20 years. In addition to clinical care capacity, laboratory research capacity was built to promote in-house translational research and biorepository for nested sub-studies to understand the HIV/AIDS epidemic as described in Table 2 and 3. Several students have used this clinical research platform to conduct research at masters', doctoral and post-doctoral level at Makerere University college of Health Sciences and collaborating academic institutions.

**Table 3:** Sub-studies to understand HIV treatment outcomes and co-infections in the IDI cohorts

Sub-study name (PI)	Methods	Key results
Cost-effectiveness of Crag screening in patients starting ART^44^ (Meya D)	The number needed to test and treat with a positive CRAG was assessed for 30-month outcomes. Cost of CRAG testing, fluconazole and amphotericin were used for the cost effectiveness analysis	Integrating CRAG screening into HIV care, specifically targeting people with CD4 cell count ≤100 cells/mL) is cost-effective in preventing disease and death; ART alone is insufficient treatment for CRAG- positive persons.
Prevalence of hepatitis B and liver enzymes elevation in patients on ART (Ocama P)^45^	In addition to liver enzymes measured in the research cohort a subset of patients who had available samples were evaluated for hepatitis B surface antigen (HBsAg) status	Hepatitis B surface antigen (HBsAg) was detected in 9%; the risk of clinically significant ART-related hepatotoxicity was low, even among HIV/HBV co- infected persons
Machine learning techniques for prediction of early virological suppres sion (Bisaso K)^46^	Multitask temporal logistic regression, patient specific survival prediction and simple logistic regression models were developed and validated using the research cohort data, and predictive performance tested on an external dataset	These models are capable of accurately predicting early virological suppression using readily available baseline demographic and clinical variables and could be used to derive a risk score for use in resource limited settings.
Plasmodium falciparum antigenaemia among asymptomatic patients on ART (Nakanjako D)^47^	Patients with sustained viral suppression were evaluated for asymptomatic malaria infection using a histidine-rich protein-2 rapid diagnostic tests in peripheral blood and microscopy to determine the parasite densities	There was low. falciparum antigenaemia after long-term successful HAART and cotrimoxazole prophylaxis. Rapid diagnostic tests for parasite-based malaria diagnosis is recommended among PLHIV that are on successful long- term anti-retroviral therapy
Immune activation and immune exhaustion among individuals with suboptimal CD4 recovery (Nakanjako D)^48^,^49^,^50^	T-cell activation and immune exhaustion (PD- 1+) were measured in patients of the research cohort patients and subcategorized in suboptimal, optimal and super-optimal. Cell proliferation was determined by carboxyfluorescein diacetate succinimidyl ester Expression of NK cell lineage markers were also measured	T cell activation and exhaustion were associated with suboptimal CD4 reconstitution despite long-term viral suppression. T-cell immune activation and exhaustion were associated with poor proliferation among suboptimal responders despite sustained viral suppression. Alteration of NK cell populations could inhibit host immune responses to infections
Persistent immune dysfunction after long- term suppressive anti- retroviral therapy (Nakanjako D)^51^,^52^,^53^	Flow cytometry assays of phenotype and function of monocytes, Natural Killer cells, and Innate Lymphoid cells among ART- treated adults who have attained CD4 counts of 500 cells/µl and over	Immune dysfunction (innate and adaptive) and inflammation persists despite recovery of CD4 counts to normal levels in peripheral blood Need for continuing search for a cure
Validation of mineral bone density using a Calcaneal ultrasound (Costa C) 54	HIV infected adults who had been on ART for ≥10 years and had undergone dual-energy x- ray absorptiometry underwent calcaneal QUS evaluation and urine sample collection	Calcaneal ultrasound showed a moderate correlation with DXA outputs. High prevalence of subclinical tubular impairment suggests expanding access to tenofovir disoproxil fumarate-sparing regimens in resource-limited settings
Bone mass density in people living with HIV on long-term anti- retroviral therapy (Mwaka E) 55	People living with HIV that had been on ART for at least 10 years had had dual X-ray absorptiometry to determine their bone mineral density. The data collected included antiretroviral drug history and behavioral risk data.	Higher body mass index was associated with a reduction in low bone mineral density of the hip and lumbar spine. Maintaining adequate body weight is important in maintaining good bone health in people on antiretroviral therapy

Crag: Cryptococcal antigen; NK: natural killers; ART: antiretroviral treatment

Subsequently, IDI investigators were able to adapt their experience to evolving research questions and attract competitive research grants from various funding agencies in USA, United Kingdom, Europe and the rest of Africa.

Using the evidence generated in these local cohorts, many IDI's scientists play key roles on the Ministry of Health advisory roles in the Paediatrics and Adult National HIV treatment committees, and these results have informed real time development, implementation and revisions of HIV/AIDS treatment and prevention guidelines. Therefore, results from studies and data within the well-characterized IDI cohorts continue to inform policy and practice in the management and prevention of HIV/AIDS.

The infrastructure of the clinic, laboratories, expert scientists, data management systems and statistics unit have positioned the institution with the agility required to respond to other emerging and re-emerging infections including the current COVID19 pandemic where IDI playing key roles in health-worker training.

### Impact

The impactful research through the HIV patient cohorts at IDI have put Makerere University at the center of the response to the HIV pandemic in the region. The capacity building and infrastructure developed as part of these cohorts, including laboratories, a robust statistics and internationally recognized scientists in the field, have prepared the institution to respond to other emerging and re-emerging infections. A case in point is the current COVID 19 pandemic where IDI is taking lead in supporting the national response to the epidemic and innovative ways of virtual training of health workers on COVID-19 management to minimize transmission and improve access to the training.

Twenty years ago, it was important to understand the early outcomes of the scale up of ART in SSA, and scientists were worried about early mortality, adherence to ART and retention. Two decades later, once established that ART scale up was an overall success promoted by good individual level adherence and creative models of care, scientists aim to understand long term HIV complications, NCDs and aging in PLWH.

In this paper we demonstrated how the IDI cohorts have informed national and international programs through dissemination not only at local level through the investigator's participation at National HIV treatment committees, but internationally by presenting at renowned Conferences and publishing in high impact journals. Additionally, since 2008 the IDI has always featured in the International Workshop on HIV and Hepatitis Observational Databases (IWHOD) which brings observational database researchers together to advance the methodology and analysis of observational data, with the IDI cohorts being among the very few from sub-Saharan Africa.

The IDI routine cohort also contributes data to the International epidemiology Databases to Evaluate AIDS (Ie-DEA) which is an international research consortium established in 2006 by the US National Institutes of Health to provide a rich resource for globally diverse HIV data^56^. The IDI routine cohort and its investigators contribute to analysis and publications at regional^57^ (East Africa) and global level^58^–^60^ where over 2.2 million people living with HIV are represented, contributed by clinical centers in 44 countries from 7 geographic regions.

We recognize that the finding from the IDI cohorts may not always be generalizable to others settings like rural clinics run by clinical officers and nurses, and generally to primary care settings. However, these models have generated evidence to inform care, policy and practice, and can be duplicated by other academic institutions in order to optimize the gains of our academic research to provide tertiary care. Most importantly we were able to evaluate the models of care implemented by the IDI cohorts and adapt them and export them to serve communities empowering lower units to continue delivering primary care rather than replacing their function.

## Conclusion

Utilizing the IDI cohort studies, we demonstrated that investment in research cohorts has earned great gains in building a critical mass of scientists through various research training initiatives. Despite the high maintenance cost, cohorts present valuable clinical care, training, and research to inform strategic response to local and global pandemics.
